# Influence of Using a Contrast-Enhanced CT Image as the Primary Image on CyberKnife Brain Radiosurgery Treatment Plans

**DOI:** 10.3389/fonc.2021.705905

**Published:** 2021-09-16

**Authors:** Jianping Zhang, Lin Wang, Benhua Xu, Miaoyun Huang, Yuangui Chen, Xiaobo Li

**Affiliations:** ^1^Department of Radiation Oncology, Fujian Medical University Union Hospital, Fuzhou, China; ^2^Fujian Medical University Union Clinical Medicine College, Fujian Medical University, Fuzhou, China; ^3^Department of Medical Imaging Technology, College of Medical Technology and Engineering, Fujian Medical University, Fuzhou, China

**Keywords:** stereotactic radiosurgery, contrast agent, dosimetry, tumor control probability, CyberKnife

## Abstract

**Background and Purpose:**

This study aimed to quantify the differences between pre- and post-contrast agent (CA) CT for CyberKnife brain SRS plans.

**Materials and Methods:**

Twenty-five patients were retrospectively analyzed. They were divided into two categories, inhomogeneous cases (13 patients) and homogeneous cases (12 patients), according to whether the tumor was close to the cavity and inhomogeneous tissues or not. The pre-CA and post-CA plans were designed and calculated using the same monitor unit and paths as those in the ray-tracing algorithm, respectively.

**Results:**

The CT number difference of tumor between pre- and post-CA was significant (on average, 24.78 ± 18.56 HU, *P*-value < 0.01). The deviation value of the target was the largest at approximately 37 HU (inhomo-) and 13 HU (homo-) (*P* < 0.01), and the values of the organs at risk (OARs) were not statistically significant (*P*-value > 0.05). However, it was not statistically significant for the dose difference between the two groups with the injection of CA (*P*-value > 0.05). The absolute effective depth difference generally remained at a level of 1 mm, but the dose difference was quitely fluctuated sometimes more than 20%. The absolute effective depth difference of the inhomo-case (0.62 mm) was larger than that of the homo-case (0.37 mm) on median, as well as the variation amplitude (*P*-value < 0.05). Moreover, the relative dose differences between the two cases were 0.38% (inhomo-) and 0.2% (homo-), respectively (*P*-value < 0.05). At the criterion of 1 mm/1%, the gamma pass rate of the homo-case (95.89%) was larger than that of the inhomo-case (93.79%). For the OARs, except for the cochlea, the two cases were almost the same (>98.85%). The tumor control probability of the target was over 99.99% before and after injection of a CA, as well as the results for the homo-case and inhomo-case.

**Conclusions:**

Considering the difference of evaluation indexes between pre- and post-CA images, we recommended plain CT to be employed as the primary image for improving the CK treatment accuracy of brain SRS, especially when the target was close to CA-sensitive OARs and cavity.

## Introduction

Stereotactic radiosurgery (SRS), such as single-fraction or hypofractionated (two to five fractions) cranial radiosurgery, is commonly used to treat various primary and metastatic brain tumors (1). Compared with other modern SRS treatment equipment, including different types of linear accelerators and Gamma Knife, the frameless image-guided radiosurgery system CyberKnife (CK) can deliver a high conformal dose to the tumor accurately and spare normal brain tissue (2–6). To make it easier and more convenient to contour the target, the patient is often injected with a contrast agent (CA) when CT scanning is performed (7). Unlike other treatment planning systems (TPS), MultiPlan needs to select only one of the CT scans as the primary image on which the 3D dose distribution is calculated. Due to limitations of the MultiPlan system, it is not convenient for oncologists to delineate the tumor by fully combining the primary simulation CT image with secondary images, such as those of enhanced CT, MRI, and PET-CT, when plain CT is chosen as the primary imaging modality. The doctors can contour the target by combining with two clearly displayed images (such as enhanced CT and MRI) at the same time, if the enhanced CT was employed as the primary image. Thus, the delineation error caused by the limitation of the MultiPlan, to some degree, is reduced. This has the potential to improve outcomes *via* disease control and to increase safety. However, dose deviation occurs because the Hounsfield unit (HU), relative electron density, and monitor unit (MU) change for the tissues containing a CA (8). As a result, the intended control dose may not be precisely delivered to the patient when treated daily without a CA. Previous studies have pointed out that the dose difference is generally less than 2% owing to the contrast agent for IMRT head and neck cases (9, 10), while it is rarely studied for CK systems. Only Kim et al. (8) reported the situation between pre- and post-CA for thoracic and abdominal tumors without brain cancers. To the best of our knowledge, a dose difference exists between tumors located in homogeneous tissues (homo-case) and those closed to inhomogeneous tissues (inhomo-case) such as a cavity. CK planning contains hundreds of beams, and the contribution to the dose from every beam is easily affected. The aim of this paper is to quantify the difference between pre-CA and post-CA brain SRS radiosurgery treatment plan, supplying evidence whether enhanced CT can be used to calculate the dose. This paper on brain tumors sought, therefore, for homo- and inhomo-cases between pre-CA and post-CA the following:

- to quantify the difference in CT values,
- to quantify the target and organ at risk (OAR) dosimetric differences,
- to quantify the gamma difference for each target and normal tissue,
- to quantify the dose and effective depth difference for each beam, and
- to quantify the tumor control probability (TCP) difference.

## Materials and Methods

Twenty-five brain cancer patients who underwent SRS or hypofractionated radiosurgery therapy at Fujian Medical University Union Hospital were randomly selected for this study. The plan difference of primary CT (pre-CA and post-CA) on which the clinical delivery plan was designed was first assessed for these patients. According to whether the tumor was close to the cavity anatomical structure or not, the patients were dichotomized into homo-case and inhomo-case. We called the tumor closed to the cavity or inhomogeneous tissues such as acoustic neuroma and pituitary tumor as inhomo-case (see **Figure S1**). For this group, many beams with dose passed through the cavity and nonuniform density structure and then focused on the tumor. For 12 patients, the tumor was surrounded by uniform density normal tissue such as most brain metastases. Almost all beams with dose focused on the tumor and did not pass through the cavity and inhomogeneous tissues; we referred to this group as the homo-case (see **Figure S2**). The patient details are listed in **Table 1**.

**Table 1:** Patient details in this study.

Characteristics	*n* = 25
Age	
Median (range)	58 (32–77)
Sex	
Male/female	11/14
Site of tumor	
Homo-case	12
Brain metastasis (lung)	8
Brain metastasis (breast)	2
Brain metastasis (esophagus)	1
Brain stem recurrence (glioblastoma)	1
Inhomo-case	13
Pituitary tumor	2
Acoustic neuroma	4
Cavernosum angioma	2
Hemangiopericytoma	1
Giant cell granulation	1
Meningioma	3

### Patient Image Acquisition

The patient was immobilized with a thermoplastic mask to cover the whole head. CT images were collected using multi-slice computed tomography (Brilliance CT, Big Bore, GE, USA) with a 1-mm slice thickness. A plain CT image without an intravenous contrast agent was scanned first, and then the patient underwent enhanced CT scanning immediately after the first scan at the same supine and head position. According to the age and cardiac output among patients, the contrast agent administration technique was individually applied. The computed tomographic angiography principle and parameter range employed in our study are listed in **Table 2**. The two sets of CTs were imported into the MultiPlan TPS of CyberKnife.

**Table 2:** The computed tomography angiography principle.

CT scan	
Voltage	120 kV
Current	400 mAs
Contrast agent	
Ultravist^®^ 300	
Volume	60 ml
Nacl	25 ml
Delay time	20-30 s
Injection flow	1.4–2.5 ml/s

### Organ Delineation and Treatment Planning

The target was delineated on the plain image, which was selected as the primary CT and corrected by combining it with magnetic resonance imaging (MRI), and the OARs were contoured on plain images by radiation oncologists according to a related clinical guide. A non-coplanar treatment plan was designed and optimized according to TG 101 to satisfy the clinical plan evaluation limitations (11).

This study aimed to quantify the total difference between pre-CA and post-CA caused by the injection of contrast agent media. Therefore, the beam set of the plain CT plan was copied to the enhanced CT image without any change. This task was performed using the CyberKnife planning system. First, the target and OARs were copied to the post-CA (enhanced) image. Because two sets of CT images were obtained at the same head and spine position, they would fuse perfectly. Second, the plan for the enhanced image was designed and saved as a phantom plan. Third, the patient QA plan was based on the corresponding phantom plan that was performed in the second step. Therefore, the beam direction, weight, and monitor units of the two plans were the same. Under this condition, the difference between the two plans based on plain and enhanced CT due to the injection of the contrast agent was accurately obtained, with the other influencing factors excluded.

### Analysis

Because CyberKnife TPS could not measure the CT value of the structure, Eclipse of Varian was employed to perform this work. The dosimetric statistics of the target and OARs were acquired using the CK TPS. The dosimetry and effective depth difference were extracted from the beam list log files, which were generated from the CK TPS and listed the affected depth and its corresponding dose (cGy) for every beam. This analysis required the reference point positions of the two types of plans to be the same.

The gamma difference for the target and all OARs between the pre-CA and post-CA plans at the criteria of 2 mm/2% and 1 mm/1% was calculated using the open-source program CERR (12, 13).

TCP was calculated in this study to evaluate the tumor control effect due to the injection of contrast agent media. As is well known, no proprietary biological model has been used to calculate TCP for single-fraction or hypofractionated radiosurgery at present. The linear-quadratic (LQ) model was accepted only for conventional fractionation radiotherapy. In this study, the dose was converted to equivalent doses of 2 Gy per fraction (EDQ_2 Gy_) based on the LQ model and the Park model to calculate the TCP (14–16). The converted formula was as follows:


(1) $$n_{2}d_{2}=n_{1}d_{1}(1+\frac{d_{1}}{\alpha /\beta })/(1+\frac{d_{2}}{\alpha /\beta })$$

The *n*_1_ fractions given with *d*_1_ Gy per fraction were converted to a second fractionation scheme with *n*_2_ fractions given with *d*_2_ Gy per fraction.


(2) $$n_{1}d_{1}=\frac{1}{\alpha D_{0}}(\frac{d_{2}n_{2}-n_{2}D_{0}\ln(\bar{n})}{1+d_{1}/(\alpha /\beta )})$$

where *D*_0_ and $\bar{n}$ described the slope and extrapolation number, respectively, of the linear part in a plot of the logarithm of survival *vs.* dose. In this paper *α* = 0.224 Gy^−1^ (17), *D*_0_ = 1.0 (16), $\bar{n}$ = 10 (16), and *α* / *β* = 10 (18). Therefore, the converted equation suggested by Wennberg was given by


(3) $$D_{{\text{EDQ}}_{2\;\text{Gy}}}=\left\{\begin{array}{l} equation\;1,\;\text{if}<5\;\text{Gy}\\ equation\;2,\;\text{if}>5\;\text{Gy} \end{array}\right\}$$

The TCP was calculated using the following logistic function (19):


(4) $$EUD={\left(\sum_{i=1}\left(v_{i}D_{i}^{a}\right)\right)}^{\frac{1}{a}}$$

(5) $$TCP=\frac{1}{1+{\left(\frac{TCD_{50}}{EUD}\right)}^{4\gamma 50}}$$

Here, EDU was the equivalent uniform dose *v_i_* and was the part of the target volume irradiated by a dose *D_i_*. Parameter *a* was a unitless model parameter equal to *a* = –9 (range: −8 to −10) (20, 21) for a tumor. *TCD*_50_ was the dose to control 50% of the tumor and *γ*_50_ was a specific parameter that describes the slope of the dose–response curve. They were published in a previous report equal to *TCD*_50_ = 50 Gy (18) and *γ*_50_ = 2 (22), respectively. Finally, the EUD-based TCP was calculated using an open-source free program (19).

## Results

**Figure 1:**
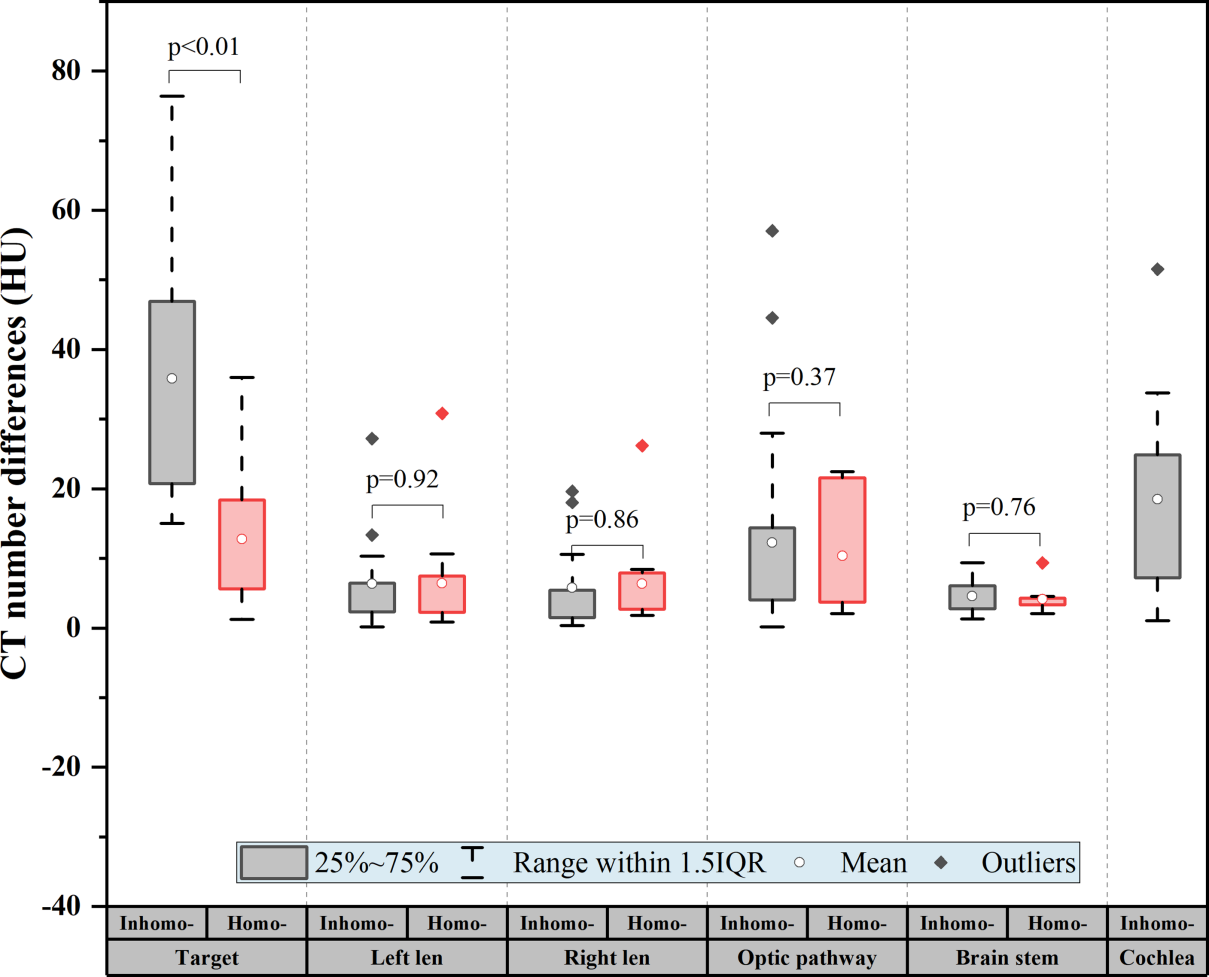
CT number difference of the target and organs at risk (OARs) for all patients. The difference = |(post-CA) − (pre-CA)|. The deviation value of the target was the largest at approximately 37 HU (inhomo-) and 13 HU (homo-) (*P* < 0.01), and the values of the OARs were not statistically significant (*P*-value > 0.05).

As shown in **Table 3**, the CT number difference [(*post*-*CA*) – (*pre*-*CA*)] of the tumor between pre- and post-CA was significant (on average, 24.78 ± 18.56 HU, *P*-value < 0.05). The HU change for OARs after the CA injection is presented in **Table 4**. The optic pathway and cochlea showed the largest change in HU, followed by the lens and brain stem. **Figure 1** shows the difference between the target and OAR CT numbers (HU) between the inhomo-case and homo-case groups due to the injection of a contrast agent. The deviation value of the target was largest at approximately 37 HU (inhomo-) and 13 HU (homo-) (*P* < 0.05), and the values of the OARs were not statistically significant (*P*-value > 0.05). Because the cochlea was not present in the homo-case group, the differences in the cochlea are listed separately. Therefore, the HU change for the inhomo-case was more easily affected by the injection of a contrast agent for tumor.

**Table 3:** CT number difference of tumor between pre- and post-CA.

CT number difference | (post-CA) − (pre-CA)|	
Max	76.38
Average	24.78
1SD	18.56
*P*-value	<0.01

**Table 4:** HU change of OARs due to CA injection.

OARs	Baseline^a^ (HU)	Max changes (HU)	Average changes (HU)
Lens	66.95	30.81	5.71 ± 6.22
Optic pathway	33.45	57.04	12.18 ± 12.52
Brain stem	30.00	9.67	4.17 ± 1.86
Cochlea	919.68	33.76	15.12 ± 10.52

a The baseline HUs for OARs were the average values in pre-CA CT scans.

**Figure 2:**
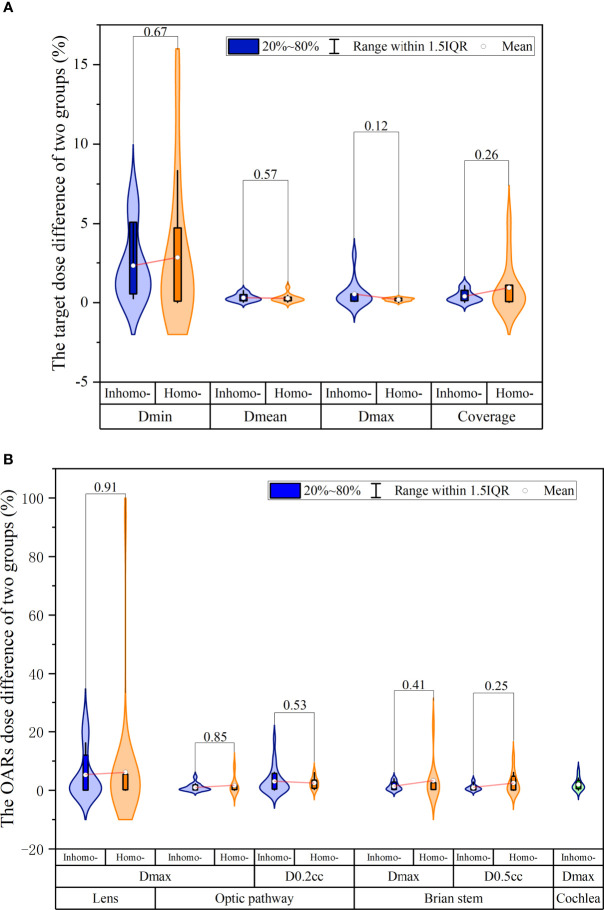
The dosimetry and effective depth difference were extracted from the beam list log files. **(A)** The target relative dose difference of the inhomo-case *vs.* homo-case; it showed that the target difference value of the homo-case in minimum dose and coverage was slightly larger than that of the inhomo-case, as well as the opposite results for mean and maximum dose. **(B)** The OAR relative dose difference of the two cases; the values of the homo-case were larger than those of the inhomo-case, except for *D*_0.2 cc_ of the optic pathway. However, they were not statistically significant.

**Table 5** presents the dosimetric differences for the target and all OARs pre- and post-CA. For the target, we knew that the difference in mean dose and maximum dose was 0.27% and 0.26% on average, respectively, and they were statistically significant (*P*-value < 0.05). Besides, we found that the other dosimetric indexes for the target and OARs were not statistically significant: the minimum dose (*P*-value = 0.08) and the coverage (*P*-value = 0.14) for the target and the maximum dose (*P*-value = 0.67) and the volume dose (*P*-value = 0.64) for OARs. **Figure 2A** shows that the target difference value of the homo-case in minimum dose and coverage was slightly larger than that of the inhomo-case (as well as the result of OARs shown in **Figure 2B**), but the opposite results for mean and maximum dose. However, it was not statistically significant for the dose difference between the two groups with the injection of CA (*P*-value > 0.05).

**Table 5:** The dosimetric difference for the target and all OARs^a^.

	Target				OARs	
	*D*_min_	*D*_mean_	*D*_max_	Coverage	*D*_max_	D*_volume_
Max	8.34%	0.76%	1.25%	3.09%	8.10%	6.23%
Average	2.07%	0.27%	0.26%	0.47%	1.38%	1.59%
1SD	2.40%	0.19%	0.25%	0.66%	1.75%	1.79%
*P*-value	0.08	<0.01	<0.01	0.14	0.67	0.64

D*_volume_ refer to D_0.2 cc_ (optic pathway) and D_0.5 cc_ (brain stem), respectively.

a The difference value = $\frac{\left|Dose(post-CA)-Dose(pre-CA)\right|}{Dose(post-CA)}\times 100\%$.

**Figure 3:**
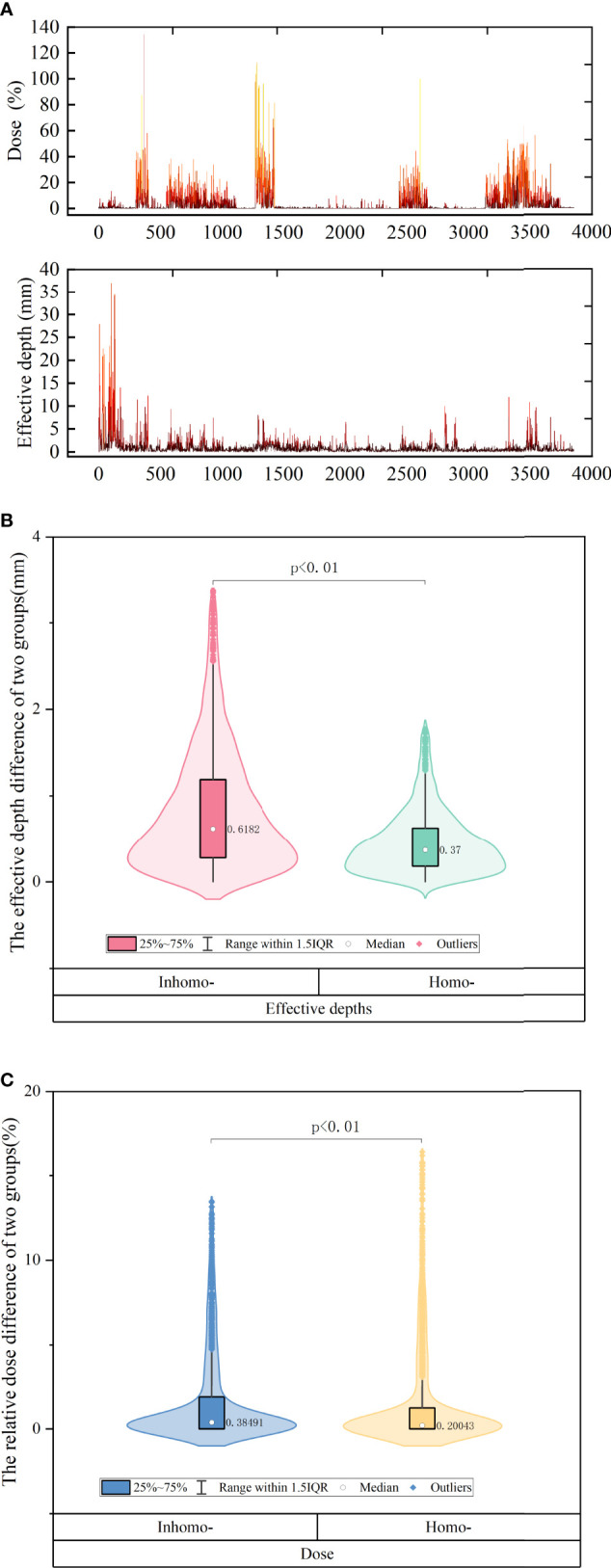
**(A)** The difference of effective depth and corresponding dose of all beams for all patients. We knew that the absolute effective depth difference generally remained at a level of 1 mm, but the dose difference was quitely fluctuated sometimes more than 20%. **(B)** The effective depth difference of inhomo-case *vs.* homo-case on median. **(C)** The relative dose of inhomo-case *vs.* homo-case on median. They were all statistically significant.

In **Figure 3A**, we knew that the absolute effective depth difference [(*post*-*CA*) – (*pre*-*CA*)] generally remained at a level of 1 mm, but the dose difference was quitely fluctuated sometimes more than 20%. **Figure 3B** shows that the absolute effective depth difference of the inhomo-case (0.62 mm) was larger than that of the homo-case (0.37 mm) on median, as well as the variation amplitude (*P*-value < 0.05). Moreover, the relative dose differences between the two cases were 0.38% (inhomo-) and 0.2% (Homo-), respectively (*P*-value < 0.05) (displayed in **Figure 3C**). In other words, the inhomo-case was more sensitive to the injection of CA when the X-rays passed through the inhomogeneous tissues.

**Figure 4:**
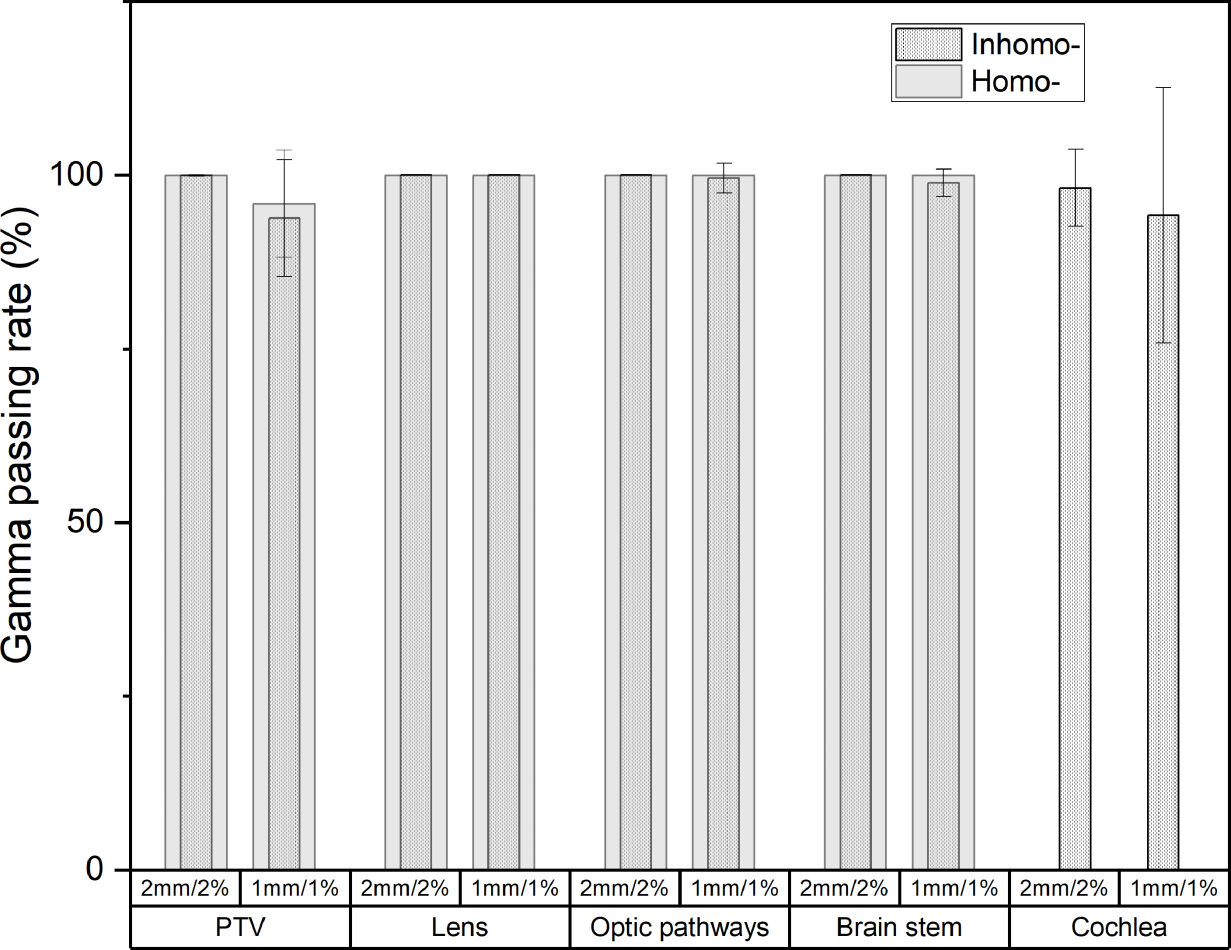
The post-CA and pre-CA 3D gamma difference of the target and all OARs for inhomo- and homo-cases. At the criterion of 1 mm/1%, the target gamma pass rate of the homo-case (95.89%) was larger than that of the inhomo-case (93.79%).

**Figure 4** shows the post-CA and pre-CA 3D gamma difference of the target and all OARs for the two cases. For the target, the gamma difference was almost the same at the criterion of 2 mm/2% for the two cases. The gamma difference of the homo-case (95.89%) was larger than that of the inhomo-case (93.79%) at the criterion of 1 mm/1%. **Supplementary Appendix A** shows that the main dose difference was the volume next to the cavity or at the edge of the tumor. For the OARs, except the cochlea, the two cases had almost the same figures (>98.85%). The cochlea differences at the criteria of 2 mm/2% and 1 mm/1% were 98.16% and 94.18%, respectively. Therefore, injection of the contrast agent affected the target more than the OARs did. This was because the target had a significant enhancement effect owing to the injection of contrast media.

According to the calculation results of this study, the TCP of the target was almost 99.99% before and after injection of a CA, as well as the results for the homo-case and inhomo-case. The contrast agent did not affect the tumor TCP.

## Discussion

For the CyberKnife treatment planning system, the primary CT image must be chosen when the clinical case plan was designed. The primary CT image was marked as A, and all the other secondary images, such as those of MRI and PET-CT, were marked as B*_i_*. Only one image can be set as the primary image. In theory, the delivery plan should be made in plain CT images due to daily treatment without CA. However, the tumor range was not too clear to contour, and the secondary images did not work well when the plain CT was employed as the primary image. If the CT image with CA was chosen as the primary image, the oncologist could synchronously delineate and review the target more easily by fusing the enhanced CT with secondary functional images. Considering the effect of the cavity and inhomogeneous tissues on the dose, the main objective of this study was to evaluate whether the enhanced CT could be chosen as the primary image for the inhomo-case and the homo-case by analyzing some indexes.

From the results (see **Table 3** and **Figure 1**), we knew that the CT value of the tumor would averagely increase 24.78 HU due to the injection of the contrast agent. The target deviation values were approximately 37 HU (inhomo-case) and 13 HU (homo-case) (*P* < 0.01). Liu et al. (9) reported that the planning target volume (PTV) CT HU change owing to the contrast agent was approximately 22.8 HU. The difference values of the OARs were not statistically significant. This was to say that the contrast agent mainly caused the increment of the tumor CT HU. The HU change for the inhomo-case was larger than that for the homo-case. This phenomenon was mainly caused by the presence of cavities. We knew that cavity invasion by the appendiceal tumor would be displayed cleanly when the contrast agent medium was injected. Unlike other commercial TPS for linac, the MultiPlan could not override the CT and density value of the structures. Therefore, more attention should be paid to the clinical case when the contrast medium was injected, especially for a target closed to the cavity.

The evaluation indexes included the minimum dose, mean dose, maximum dose, and coverage, reflecting the fact that the target dosimetry changed with the injection of CA. The difference values for *D*_min_, *D*_mean_, *D*_max_, and coverage were 2.07% ± 2.40% (max 8.34%, *P*-value = 0.08), 0.27% ± 0.19% (max 0.76%, *P*-value < 0.01), 0.26% ± 0.25% (max 1.25%, *P*-value < 0.01), and 0.47% ± 0.66% (max 3.09%, *P*-value = 0.14). For SRS radiotherapy, we usually could not accept the error exceeding 1% according to the TG 101 report. **Figures 2A, B** show the tumor and OAR dose discrepancy between inhomo- and homo-case with injection of CA, but it was not statistically significant. This conclusion was in accordance with previous studies (9, 10). From this point of view, the cavity and inhomogeneous tissues did not have a significant influence on the total dose of the target and OARs. However, CA had a noticeable effect on the dose of the target. Therefore, post-CE CT should be carefully used in CK treatment planning, especially when the beams passed through the cavity and inhomogeneous tissues.

Non-coplanar and multibeams of CK had special advantages compared with other radiotherapy equipment. They delivered a high conformal dose to the tumor and spared normal tissues as much as possible. A deliverable clinical SRS plan included approximately 200 beams. In this study, we explored the effective depths and corresponding dose differences for each beam. **Figure 3A** reflects that the absolute effective depth difference generally remained at a level of 1 mm, but the dose difference was quitely fluctuated sometimes more than 20%. It was noted that the effective depth and corresponding dose for beams were obviously influenced by CA. As shown in **Figure 3B**, the absolute effective depth difference of the inhomo-case (0.62 mm) was larger than that of the homo-case (0.37 mm), as well as the variation amplitude. The change values for the homo-case were more stable than those for the inhomo-case. Moreover, the relative dose differences between the two cases were 0.38% (inhomo-) and 0.2% (homo-), respectively (*P*-value < 0.05) (**Figure 3C**). This result agreed with previous studies (<1%) (10, 23, 24). The cavity and contrast media (growing the CT value) increased the dose, but there was a slight contribution to the effective depth. In other words, the dose was more sensitive to the variation of the effective depth for the homo-case. This was because the dose was more easily deposited when the X-rays passed through the homogeneous tissues. This was the reason that the evaluation index value of the inhomo-case was slightly less than that of the homo-case.

The post-CA and pre-CA 3D gamma differences of the target were almost the same at the criterion of 2 mm/2% for the two cases. At the criterion of 1 mm/1%, the gamma pass rate of the homo-case (95.89%) was larger than that of the inhomo-case (93.79%). For the OARs, except for the cochlea, the two cases were almost the same (>98.85%). The cochlea differences at the criteria of 2 mm/2% and 1 mm/1% were 98.16% and 94.18%, respectively. The **Supplementary Appendix A** shows that the main dose difference was the volume next to the cavity or at the edge of the tumor. This indicated that the dose close to the cavity and the tumor edge was more easily affected by external factors. Therefore, injection of the contrast agent affected the target more than it did the OARs. This was because the target had a significant enhancement effect owing to the injection of contrast media. From the above, we should properly increase the margin of the PTV (for the homo-case), especially in the direction of the cavity (for the inhomo-case) to prevent the lack of dose when we employ the post-CA image as the primary CT image.

Finally, according to the calculation results of this study, the TCP of the target was over 99.99% before and after injection of CA, both for the homo-case and inhomo-case. The contrast agent would not affect the TCP of the tumor, regardless of which CT was selected as the primary planning image.

## Conclusion

In conclusion, our study quantified the plan differences between pre-CA and post-CA images, as well as the difference for the two groups. There was a significant statistical difference in the CT value, dosimetry, gamma passing rate, effective depth, and dose, especially if a target was positioned where many beams passed through the cavity and inhomogeneous tissues. Considering the difference of evaluation indexes between pre- and post-CA images, we recommended plain CT to be employed as the primary image for improving the CK treatment accuracy of brain SRS, especially when the target was close to CA-sensitive OARs and cavity.

## Limitation

The potential limitation of this study was the small data set of the inhomo-case. The reason for this was that patients who underwent SRS radiotherapy in our center mainly were brain metastases cases, and the tumors were usually not close to a cavity. It may be subject to biases and may have influenced the *P*-value of the resulting data. However, the result of the study basically reflected the rule that there was significant statistical difference in the CT value, dosimetry, gamma passing rate, effective depth, and dose, especially if a target was positioned where many beams passed through the cavity and inhomogeneous tissues. We will supplement the data set in our future study to further verify the conclusion obtained by our research.

## Data Availability Statement

The original contributions presented in the study are included in the article/**Supplementary Material**. Further inquiries can be directed to the corresponding authors.

## Author Contributions

JZ and LW drafted the manuscript and worked on the conception, design, and interpretation of data. YC and MH designed and reviewed the treatment plans. BX and XL reviewed the data analysis. All authors contributed to the article and approved the submitted version.

## Funding

JZ is funded by Fujian Education Department Young and Middle-Aged Teacher Education Research Project of 2020 (JAT200165). BX is funded by Joint Funds for the Innovation of Science and Technology, Fujian Province (2017Y9061). YC is funded by Joint Funds for the Innovation of Science and Technology, Fujian Province (2018Y9001). XL is funded by Fujian Science and Technology Innovation Joint Fund Project (2018Y9049) and Fujian Provincial Department of Finance Project (2018B055). This work was funded by the above grants.

## Conflict of Interest

The authors declare that the research was conducted in the absence of any commercial or financial relationships that could be construed as a potential conflict of interest.

## Publisher’s Note

All claims expressed in this article are solely those of the authors and do not necessarily represent those of their affiliated organizations, or those of the publisher, the editors and the reviewers. Any product that may be evaluated in this article, or claim that may be made by its manufacturer, is not guaranteed or endorsed by the publisher.

## Supplementary Material

Supplementary Figure 1 The schematic plot of inhomo-case definition. **(A)** Coronal and **(B)** sagittal direction of generic anatomy showing the approximate path of each beam.

Supplementary Figure 2 The schematic plot of homo-case definition. **(A)** Coronal and **(B)** sagittal direction of generic anatomy showing the approximate path of each beam. The colors pink, green, and aqua represented the tumor, cavity, and beam, respectively.

Supplementary Appendix A The target gamma difference for each patient.
