# Supplementary figures and images for: Influence of Using a Contrast-Enhanced CT Image as the Primary Image on CyberKnife Brain Radiosurgery Treatment Plans
Source: Front Oncol. 2021 Sep 16;11:705905. doi: 10.3389/fonc.2021.705905 (PMC8483719; doi:10.3389/fonc.2021.705905)

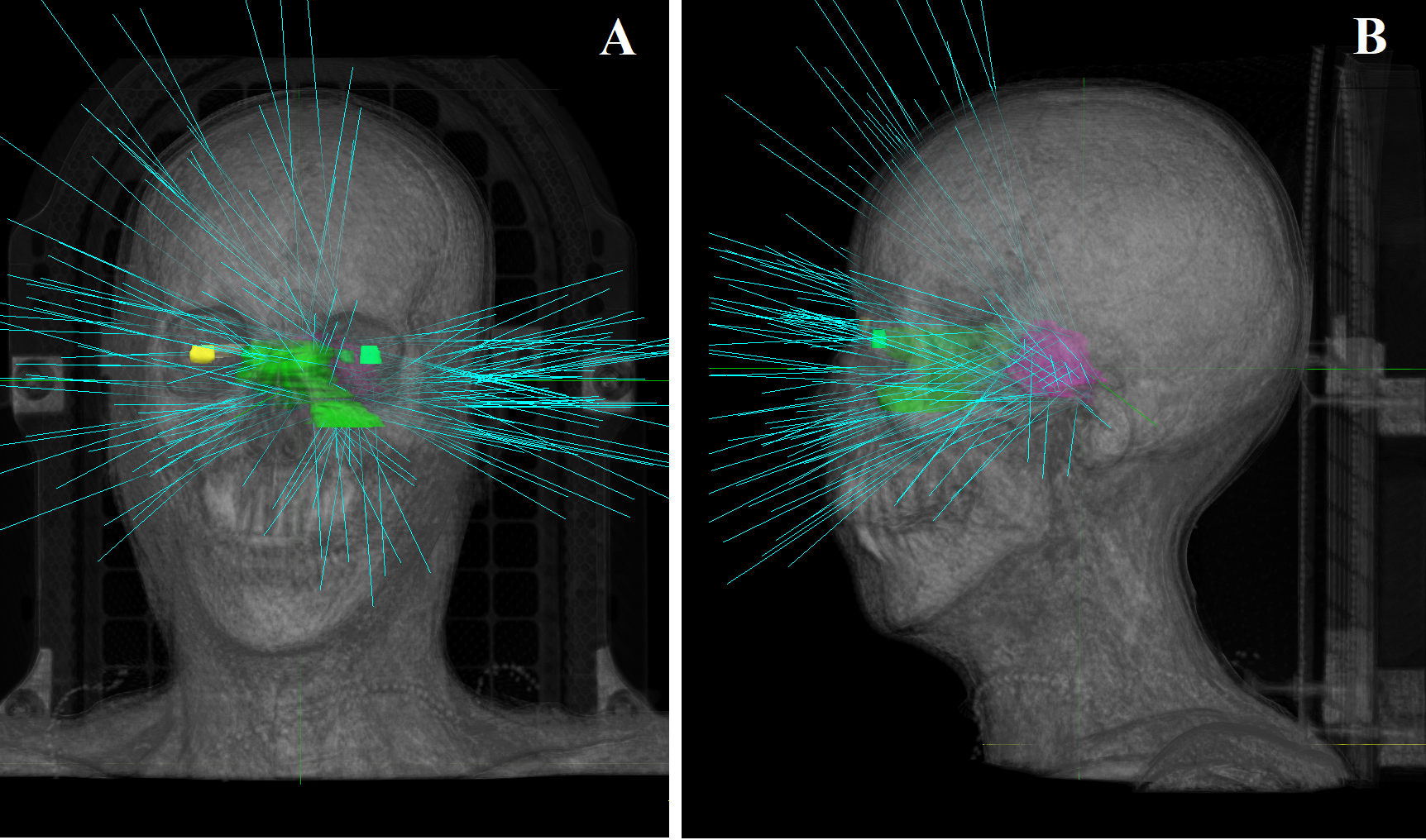

Supplement: Supplementary Figure 1 — The schematic plot of inhomo-case definition. (A) Coronal and (B) sagittal direction of generic anatomy showing the approximate path of each beam. [file Image_1.tif]

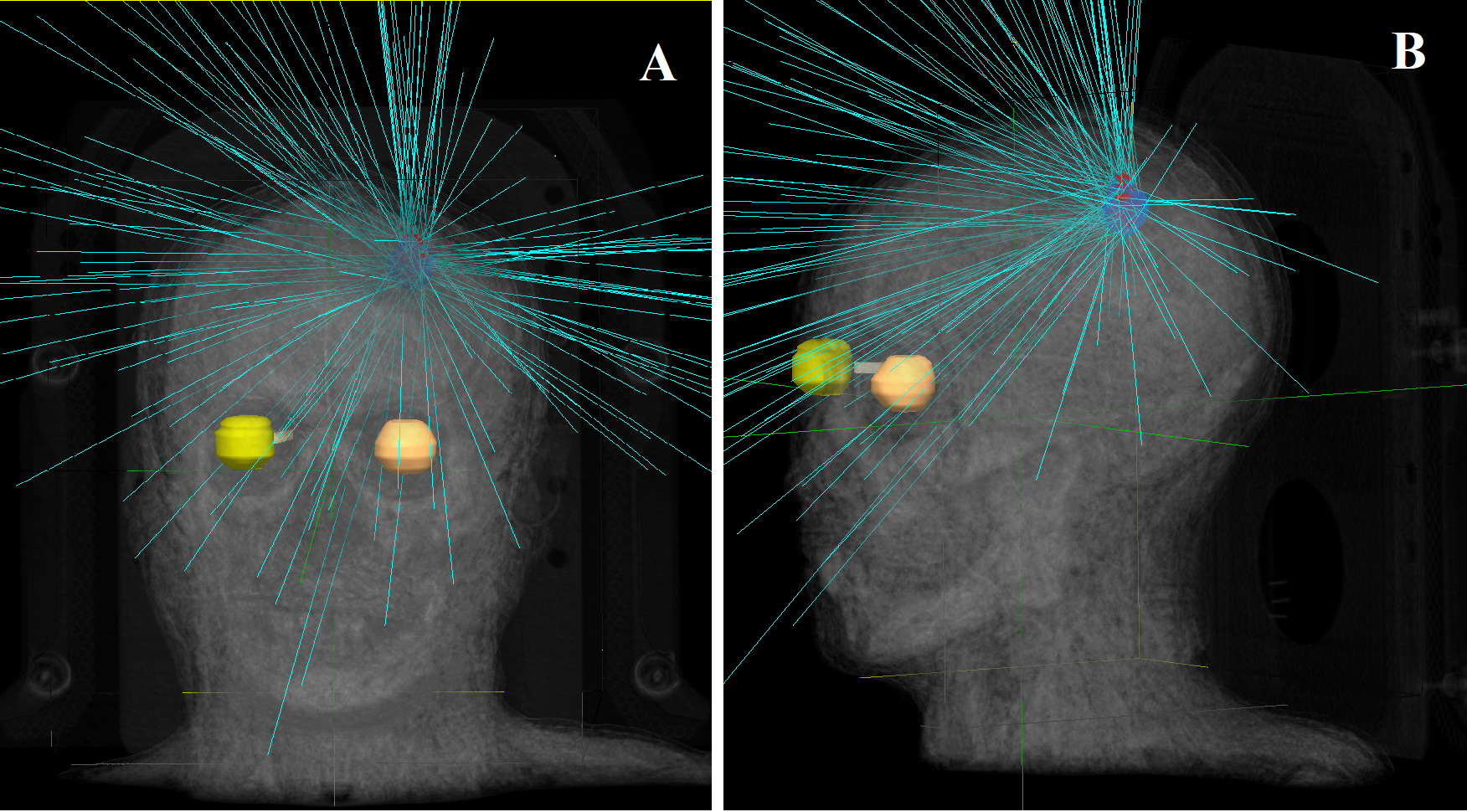

Supplement: Supplementary Figure 2 — The schematic plot of homo-case definition. (A) Coronal and (B) sagittal direction of generic anatomy showing the approximate path of each beam. The colors pink, green, and aqua represented the tumor, cavity, and beam, respectively. [file Image_2.tif]

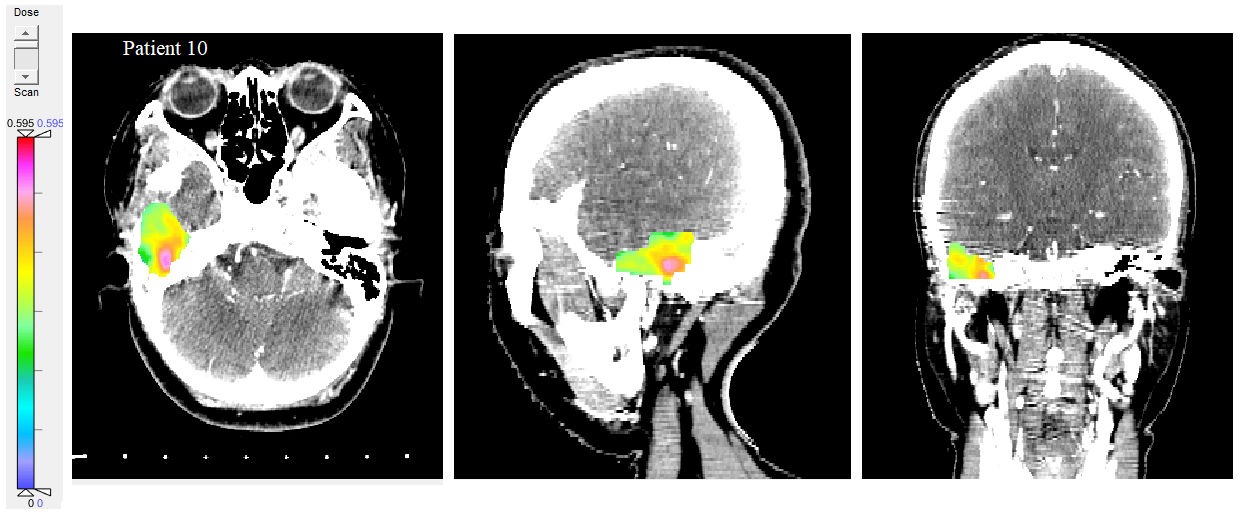

Supplement: Supplementary Appendix A — The target gamma difference for each patient. [file DataSheet_1.zip › Appendix A The target gamma difference for each patient/Patient 10-1.tif]

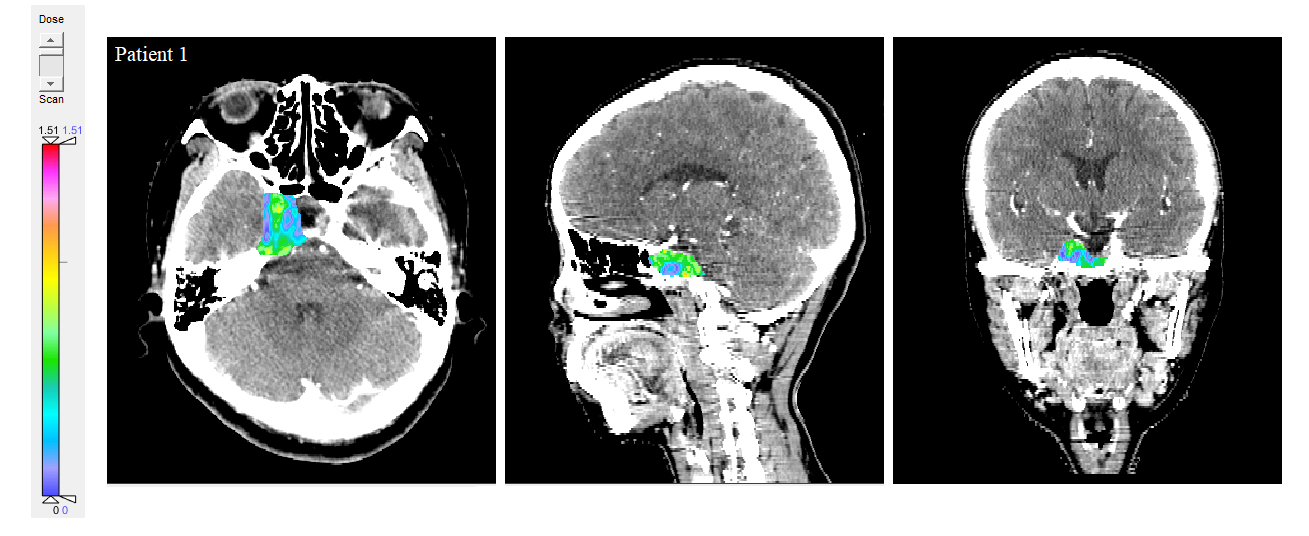

Supplement: Supplementary Appendix A — The target gamma difference for each patient. [file DataSheet_1.zip › Appendix A The target gamma difference for each patient/Patient 1-1.tif]

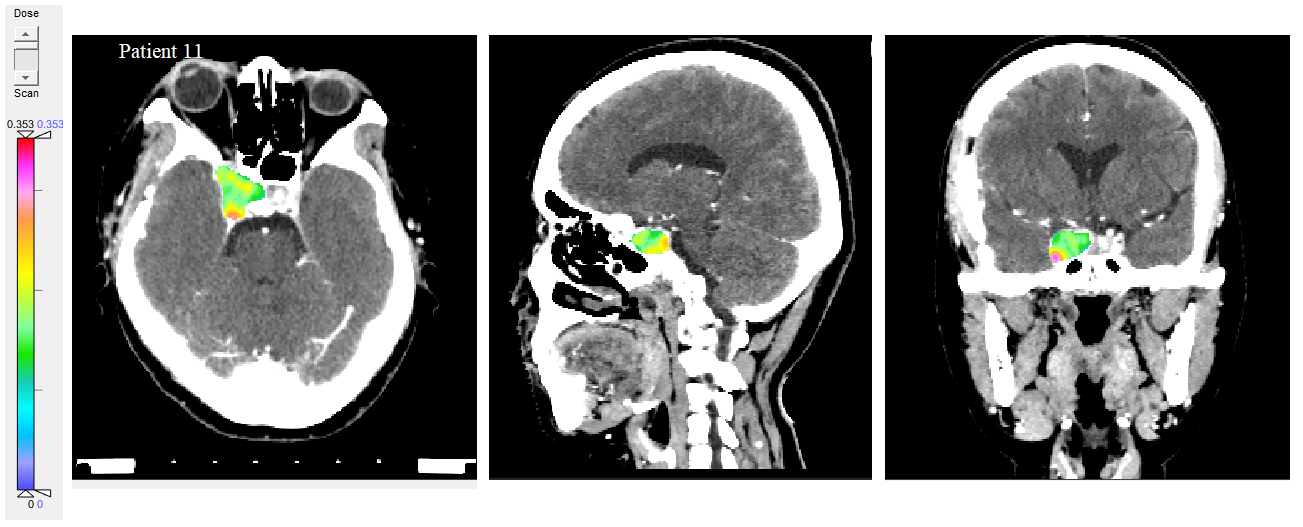

Supplement: Supplementary Appendix A — The target gamma difference for each patient. [file DataSheet_1.zip › Appendix A The target gamma difference for each patient/Patient 11-1.tif]

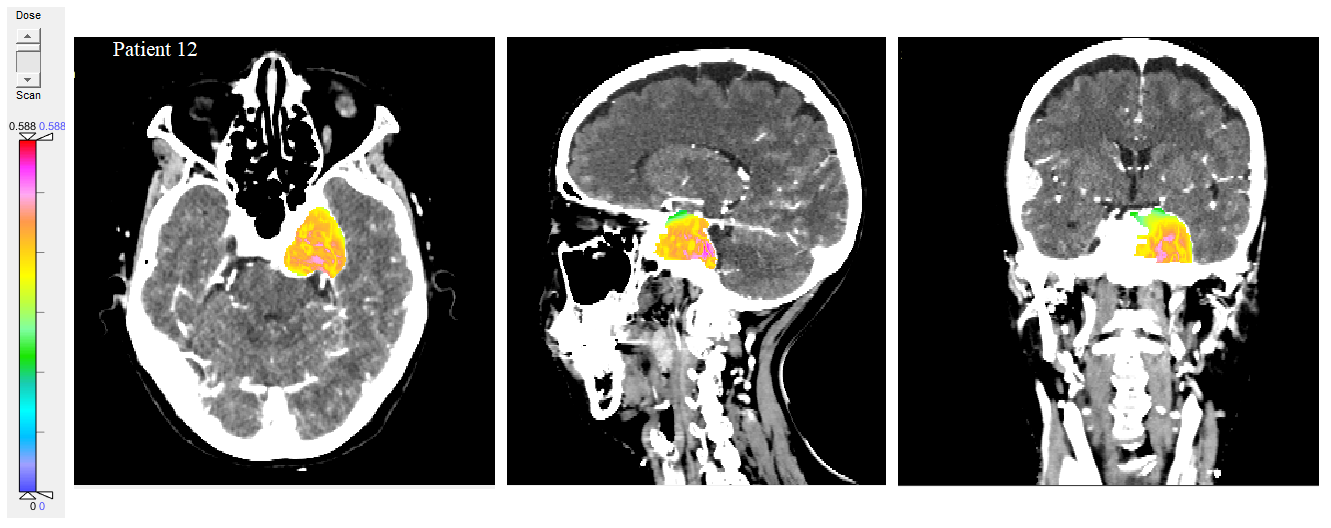

Supplement: Supplementary Appendix A — The target gamma difference for each patient. [file DataSheet_1.zip › Appendix A The target gamma difference for each patient/Patient 12-1.tif]

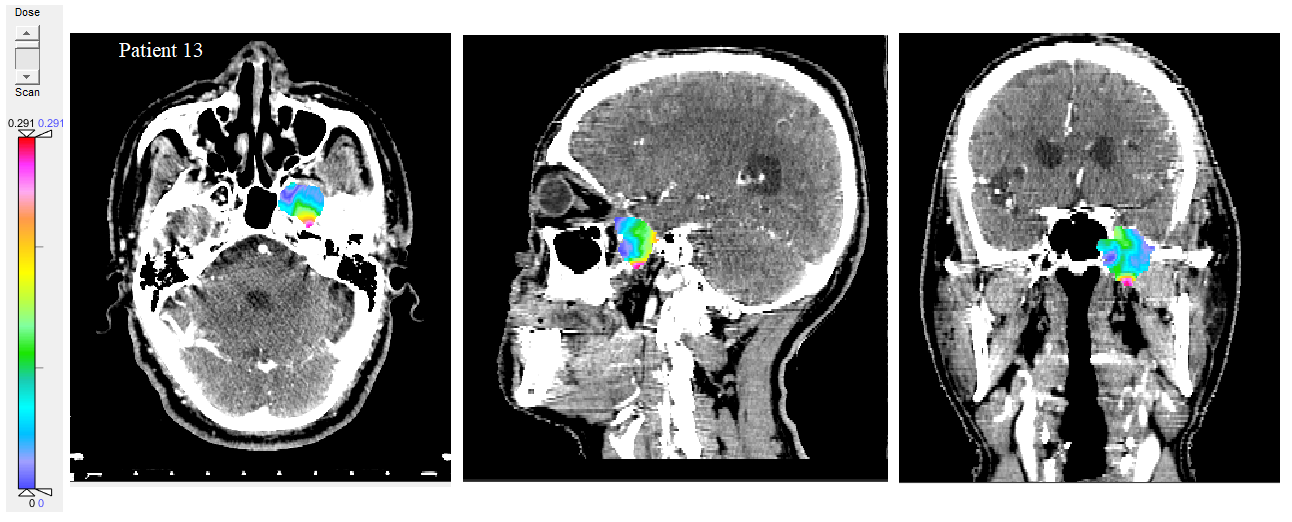

Supplement: Supplementary Appendix A — The target gamma difference for each patient. [file DataSheet_1.zip › Appendix A The target gamma difference for each patient/Patient 13-1.tif]

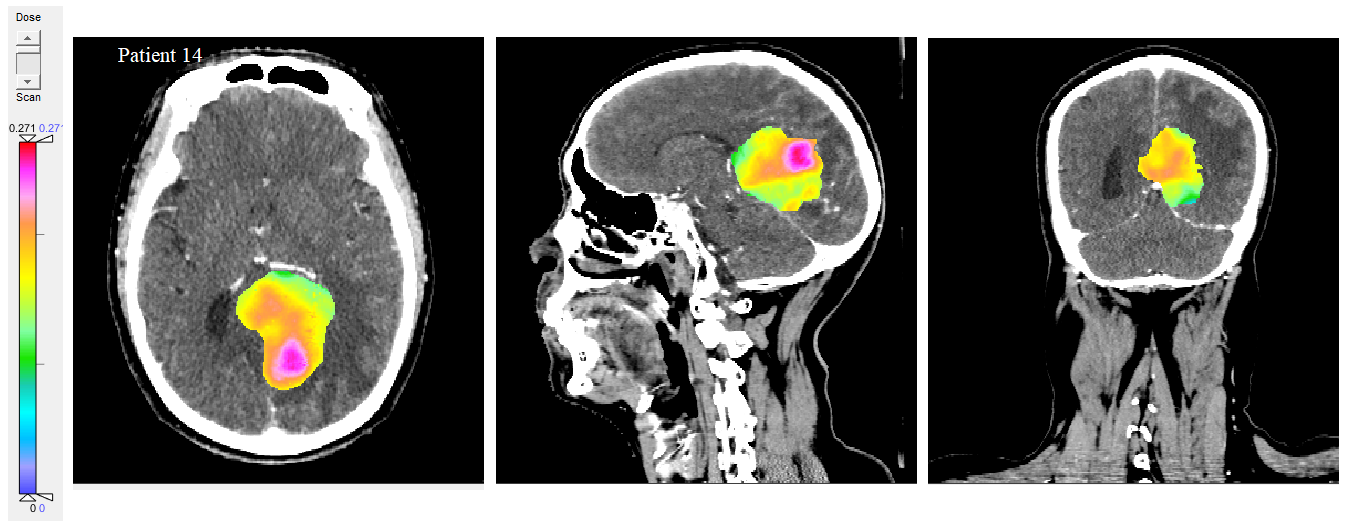

Supplement: Supplementary Appendix A — The target gamma difference for each patient. [file DataSheet_1.zip › Appendix A The target gamma difference for each patient/Patient 14-1.tif]

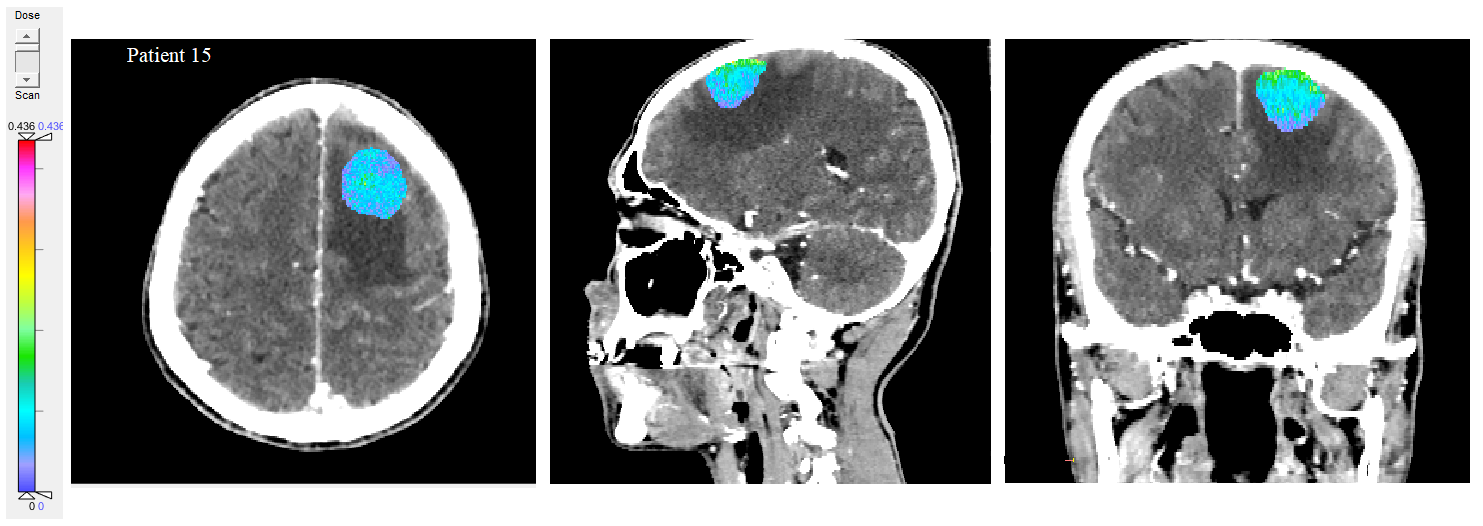

Supplement: Supplementary Appendix A — The target gamma difference for each patient. [file DataSheet_1.zip › Appendix A The target gamma difference for each patient/Patient 15-1.tif]

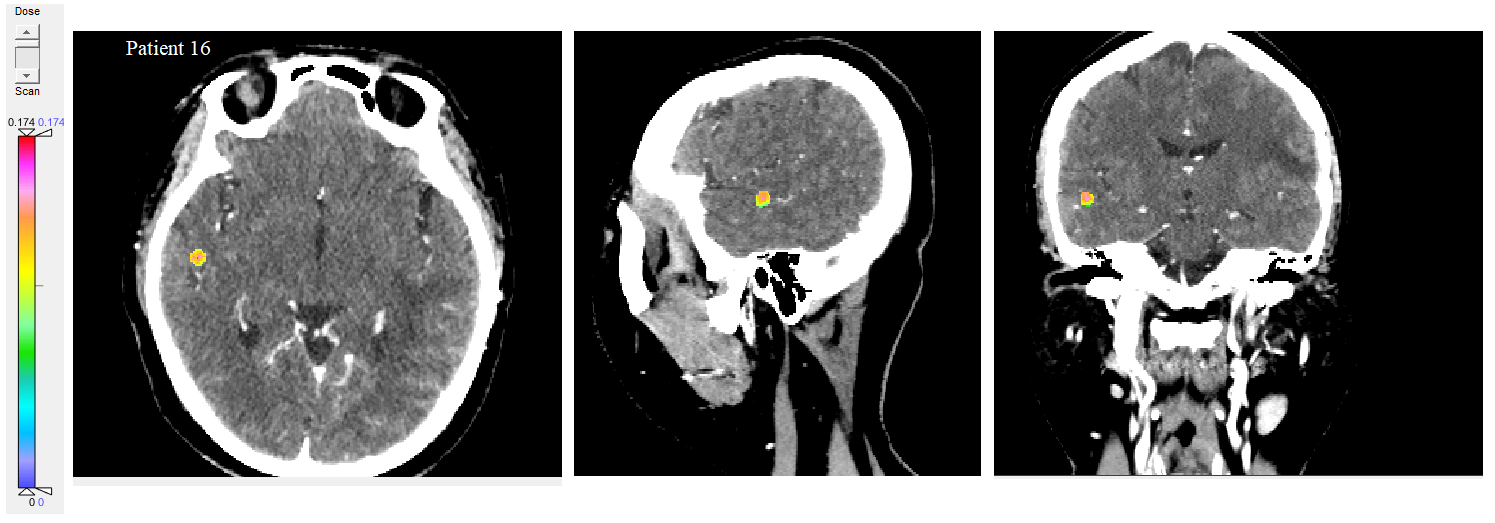

Supplement: Supplementary Appendix A — The target gamma difference for each patient. [file DataSheet_1.zip › Appendix A The target gamma difference for each patient/Patient 16-1.tif]

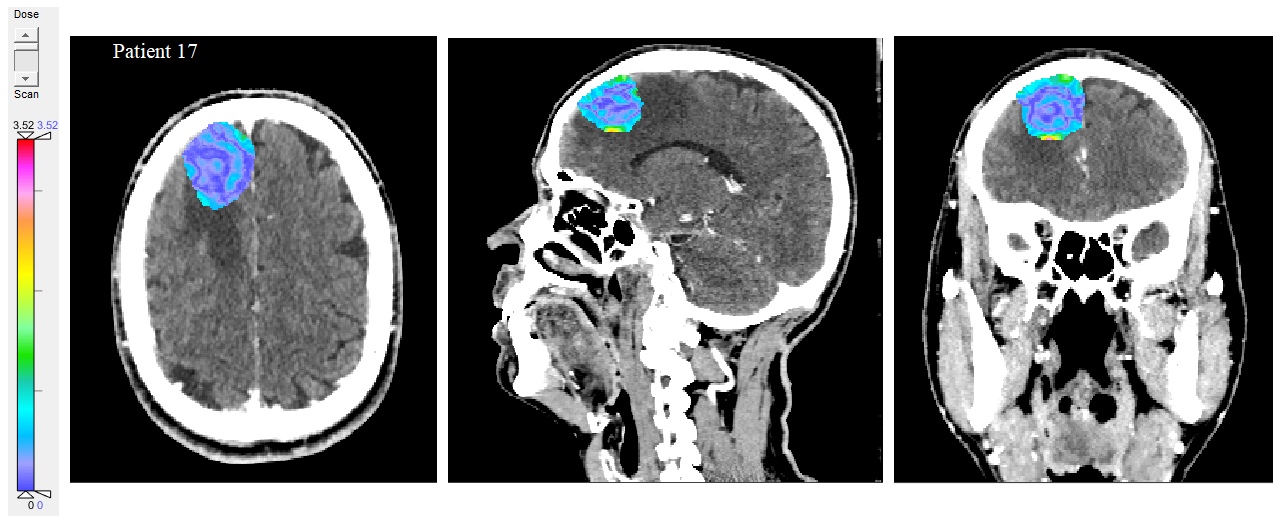

Supplement: Supplementary Appendix A — The target gamma difference for each patient. [file DataSheet_1.zip › Appendix A The target gamma difference for each patient/Patient 17-1.tif]

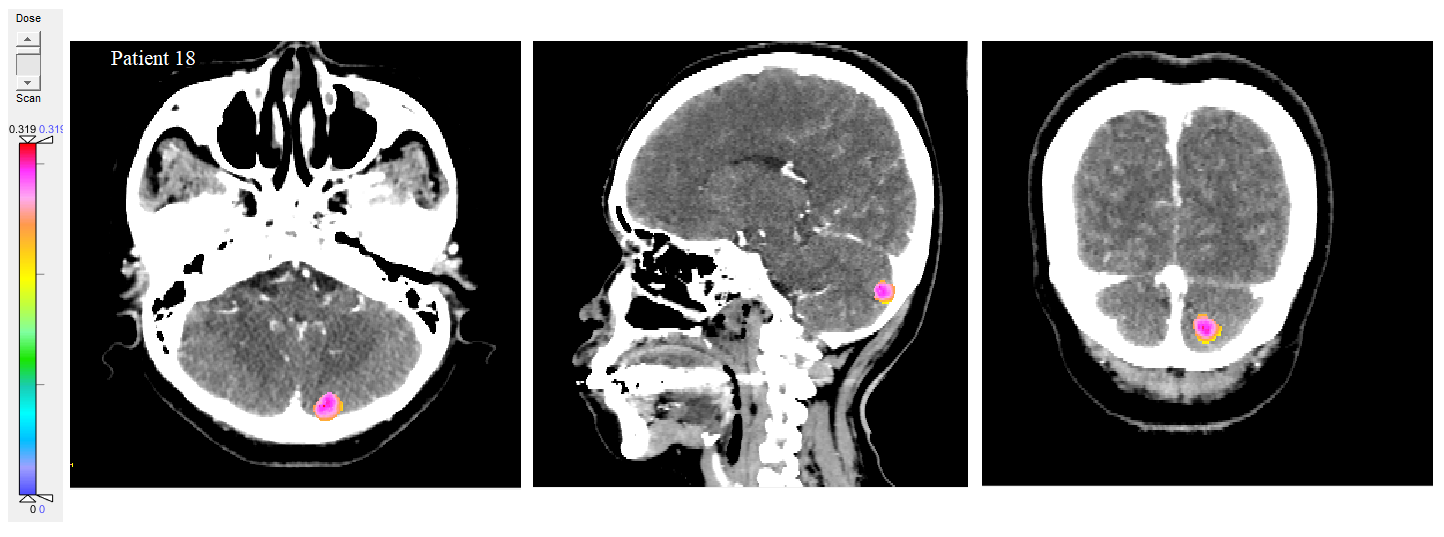

Supplement: Supplementary Appendix A — The target gamma difference for each patient. [file DataSheet_1.zip › Appendix A The target gamma difference for each patient/Patient 18-1.tif]

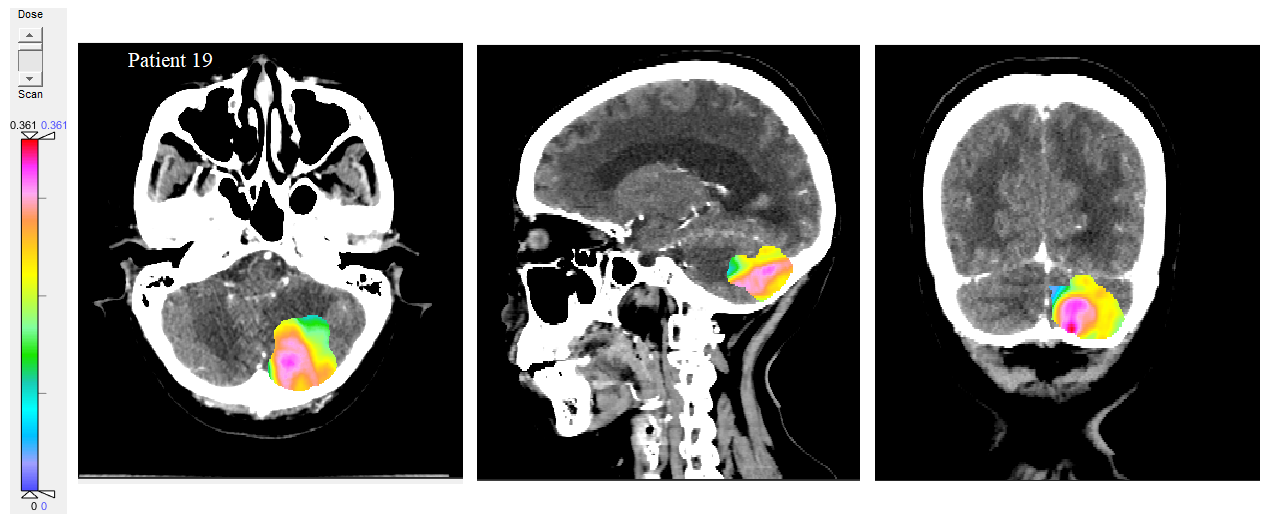

Supplement: Supplementary Appendix A — The target gamma difference for each patient. [file DataSheet_1.zip › Appendix A The target gamma difference for each patient/Patient 19-1.tif]

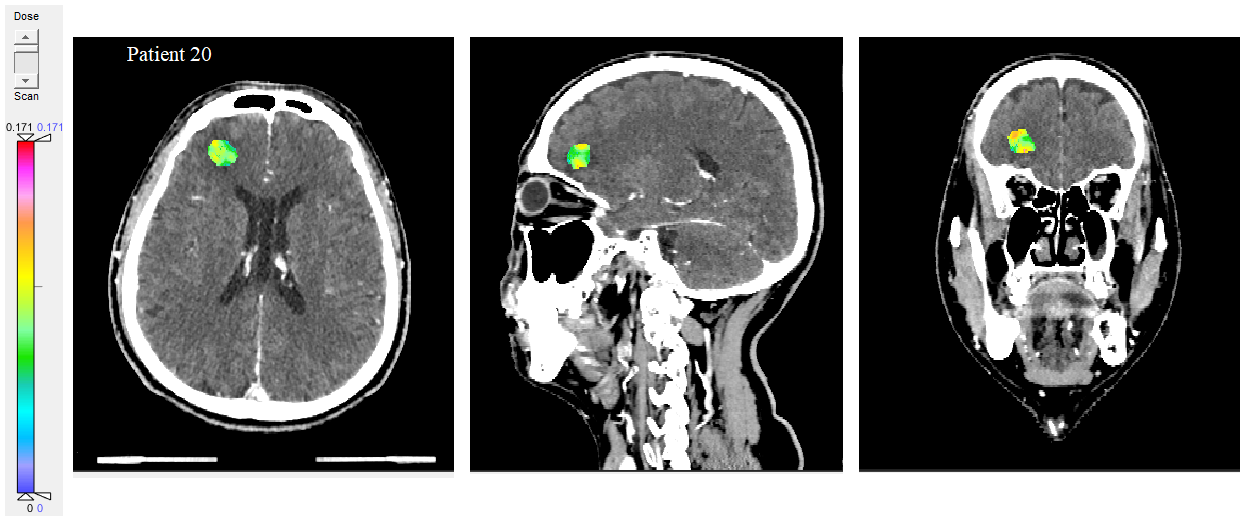

Supplement: Supplementary Appendix A — The target gamma difference for each patient. [file DataSheet_1.zip › Appendix A The target gamma difference for each patient/Patient 20-1.tif]

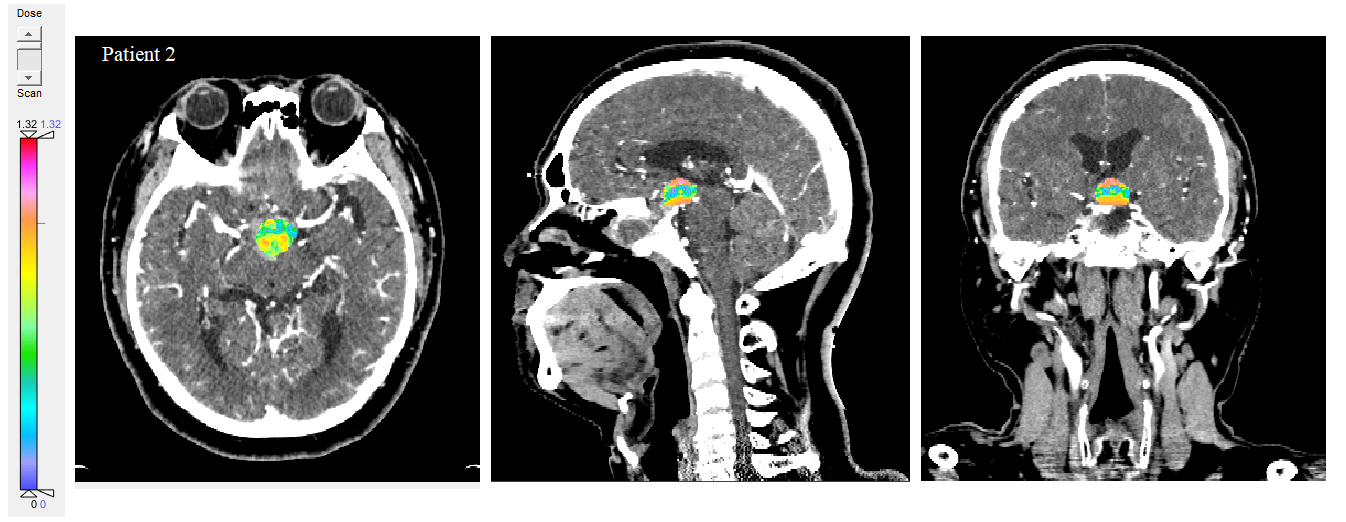

Supplement: Supplementary Appendix A — The target gamma difference for each patient. [file DataSheet_1.zip › Appendix A The target gamma difference for each patient/Patient 2-1.tif]

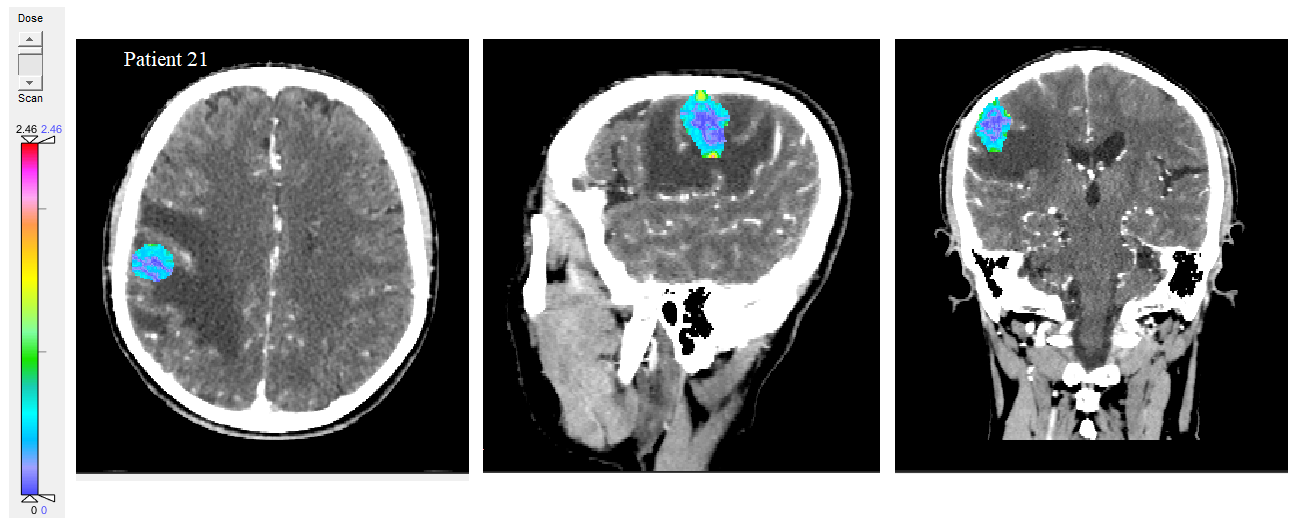

Supplement: Supplementary Appendix A — The target gamma difference for each patient. [file DataSheet_1.zip › Appendix A The target gamma difference for each patient/Patient 21-1.tif]

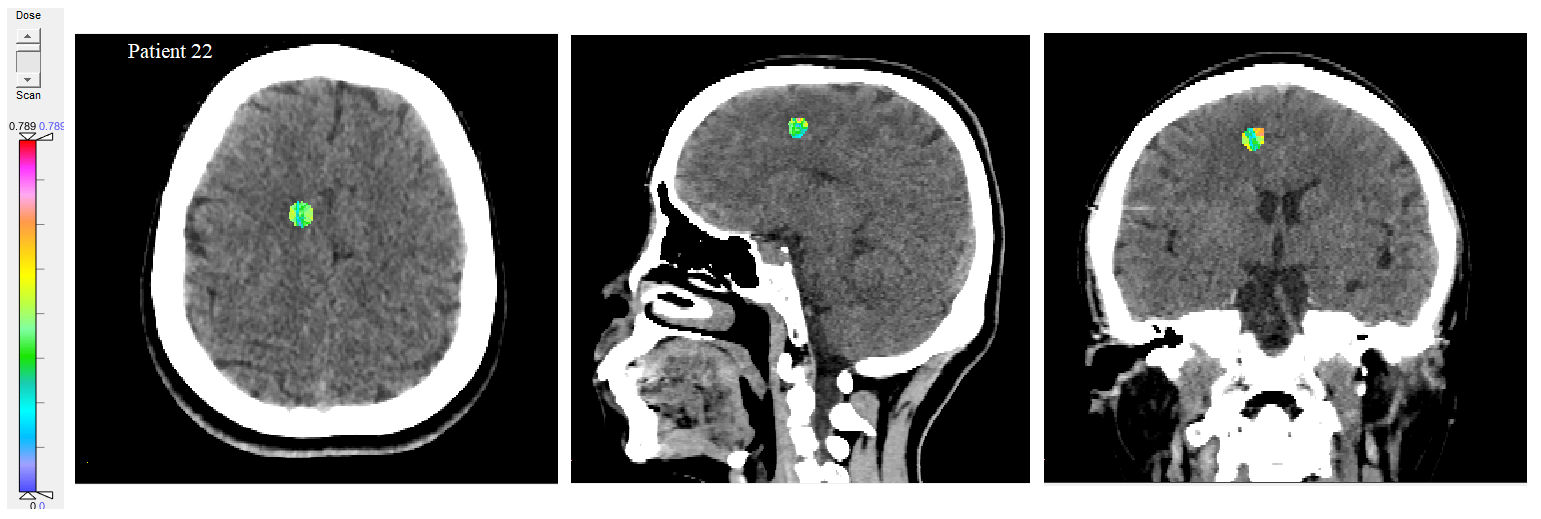

Supplement: Supplementary Appendix A — The target gamma difference for each patient. [file DataSheet_1.zip › Appendix A The target gamma difference for each patient/Patient 22-1.tif]

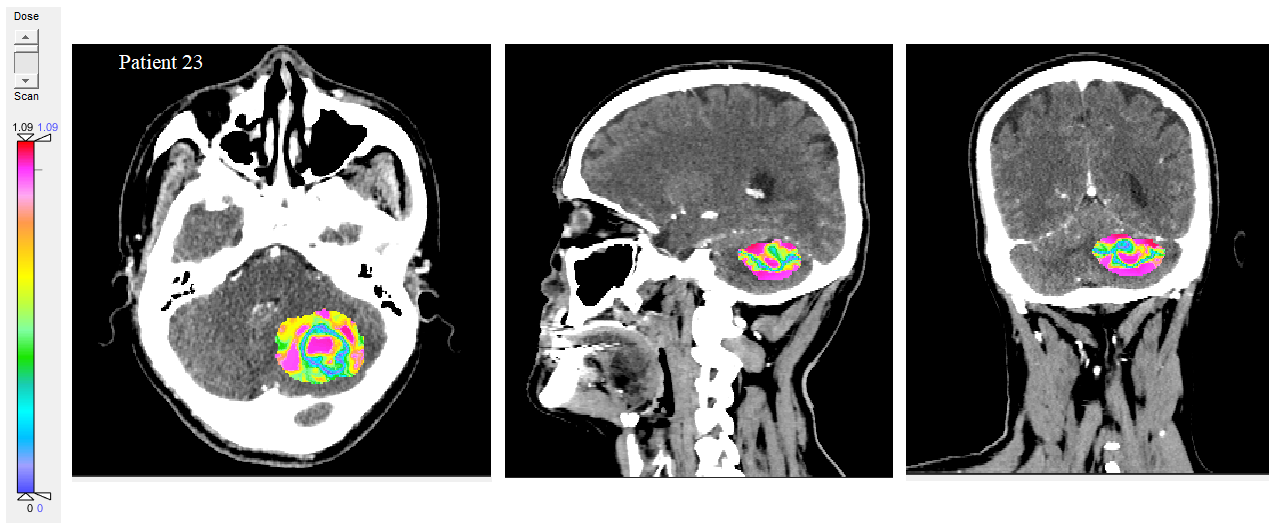

Supplement: Supplementary Appendix A — The target gamma difference for each patient. [file DataSheet_1.zip › Appendix A The target gamma difference for each patient/Patient 23-1.tif]

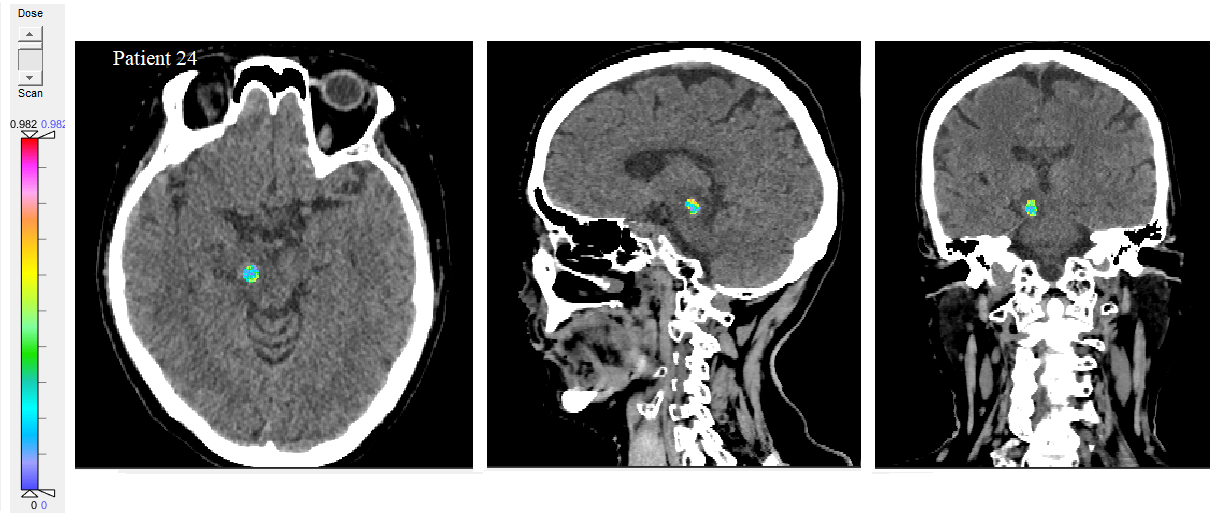

Supplement: Supplementary Appendix A — The target gamma difference for each patient. [file DataSheet_1.zip › Appendix A The target gamma difference for each patient/Patient 24-1.tif]

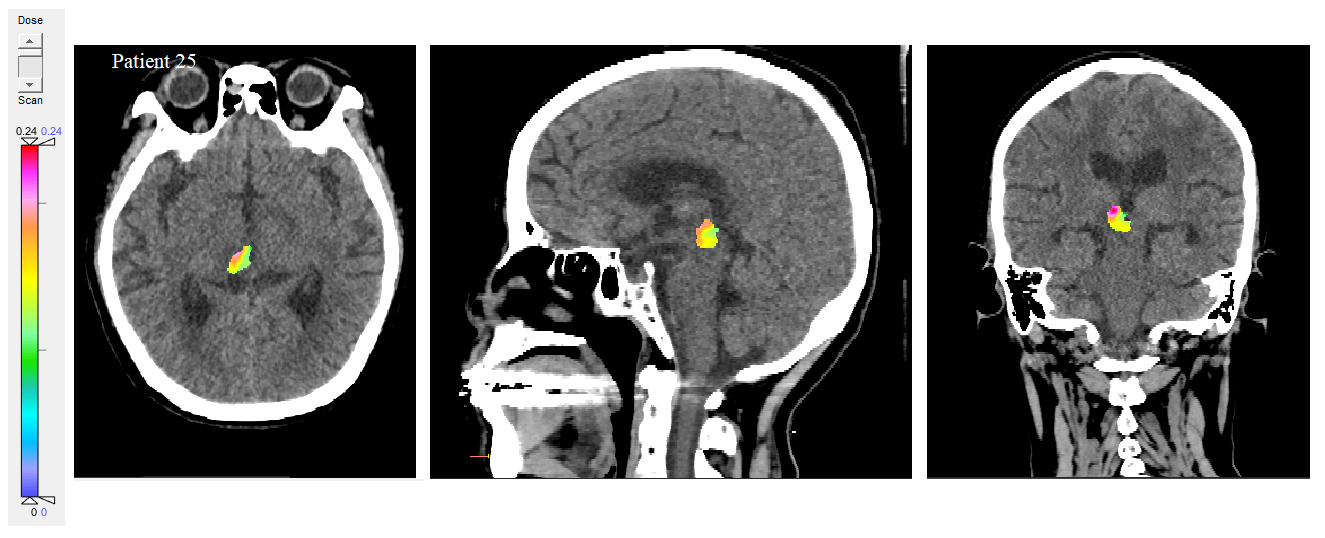

Supplement: Supplementary Appendix A — The target gamma difference for each patient. [file DataSheet_1.zip › Appendix A The target gamma difference for each patient/Patient 25-1.tif]

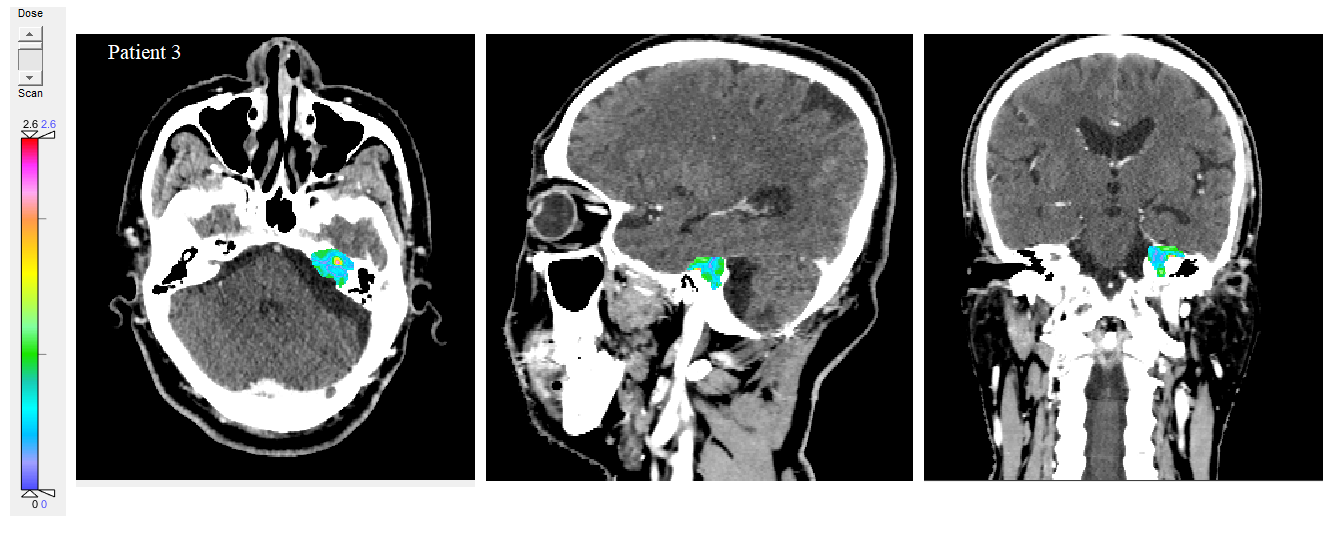

Supplement: Supplementary Appendix A — The target gamma difference for each patient. [file DataSheet_1.zip › Appendix A The target gamma difference for each patient/Patient 3-1.tif]

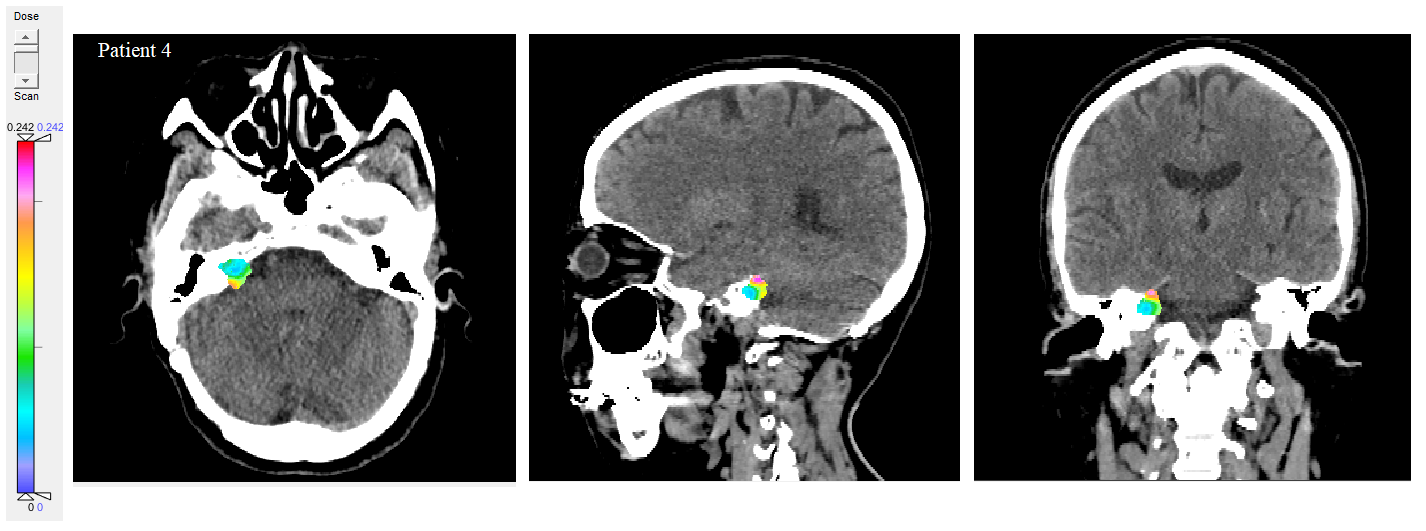

Supplement: Supplementary Appendix A — The target gamma difference for each patient. [file DataSheet_1.zip › Appendix A The target gamma difference for each patient/Patient 4-1.tif]

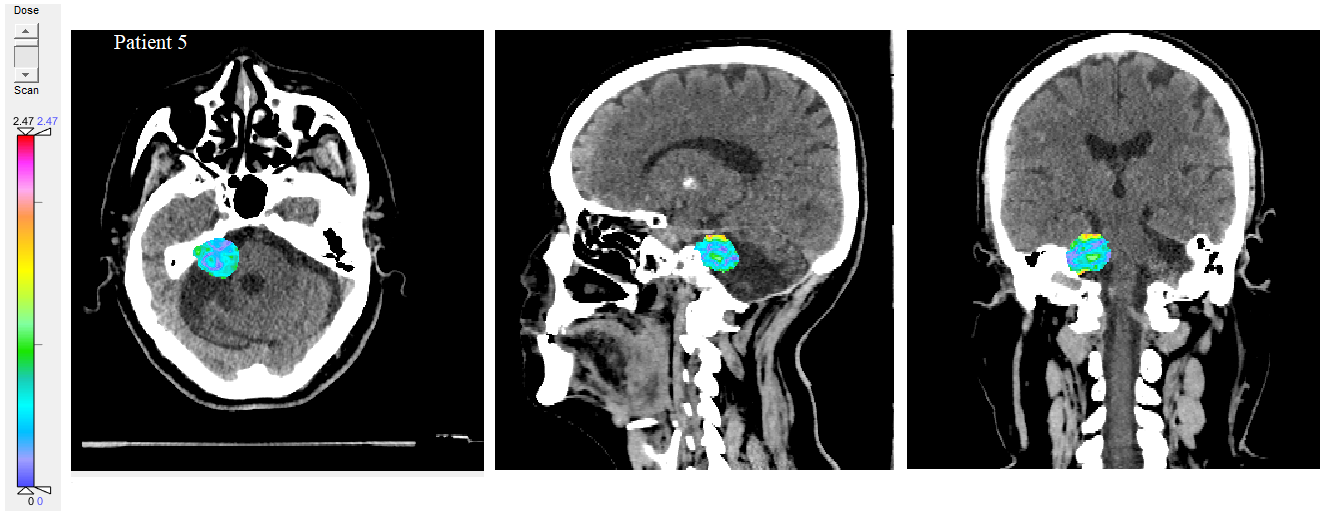

Supplement: Supplementary Appendix A — The target gamma difference for each patient. [file DataSheet_1.zip › Appendix A The target gamma difference for each patient/Patient 5-1.tif]

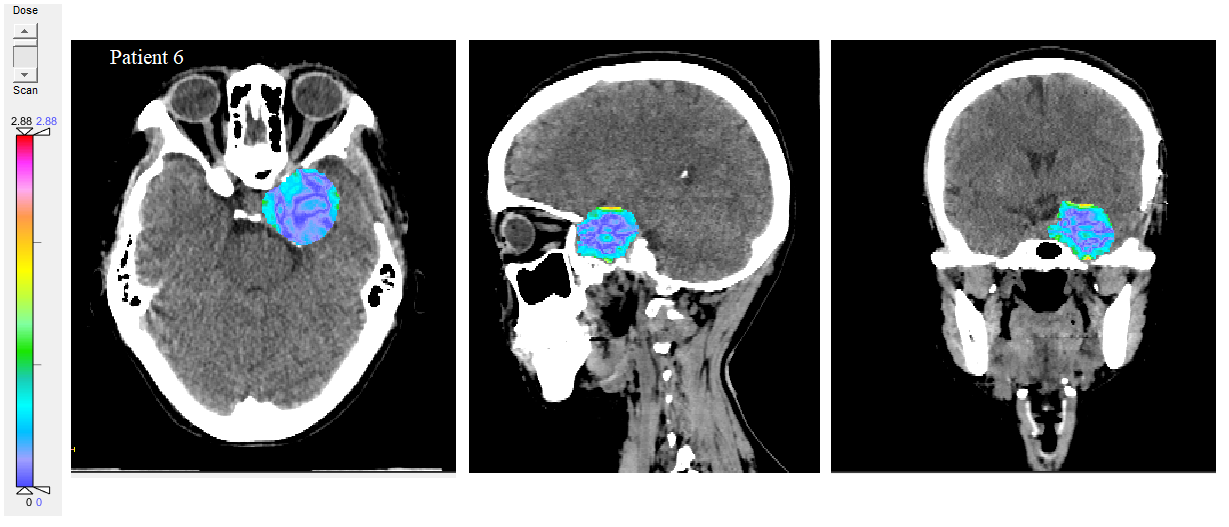

Supplement: Supplementary Appendix A — The target gamma difference for each patient. [file DataSheet_1.zip › Appendix A The target gamma difference for each patient/Patient 6-1.tif]

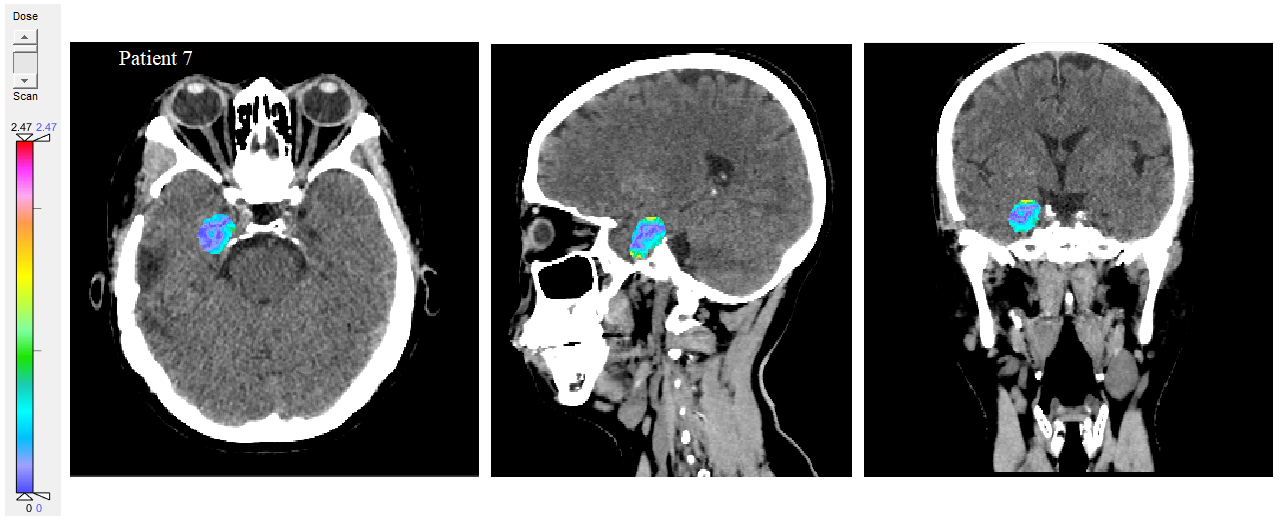

Supplement: Supplementary Appendix A — The target gamma difference for each patient. [file DataSheet_1.zip › Appendix A The target gamma difference for each patient/Patient 7-1.tif]

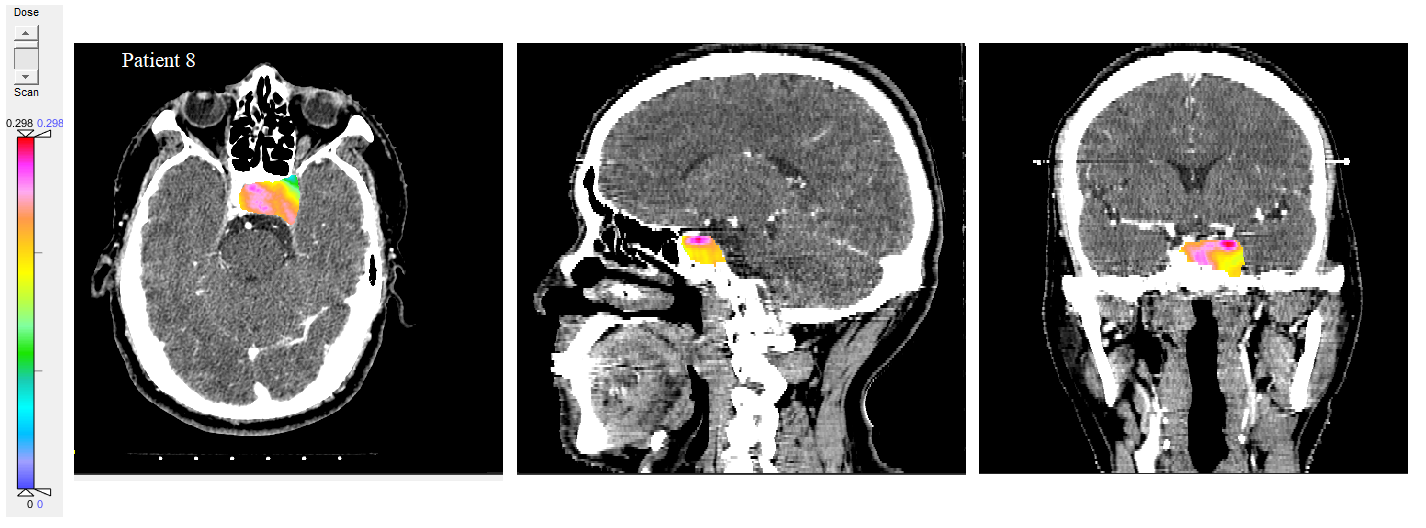

Supplement: Supplementary Appendix A — The target gamma difference for each patient. [file DataSheet_1.zip › Appendix A The target gamma difference for each patient/Patient 8-1.tif]

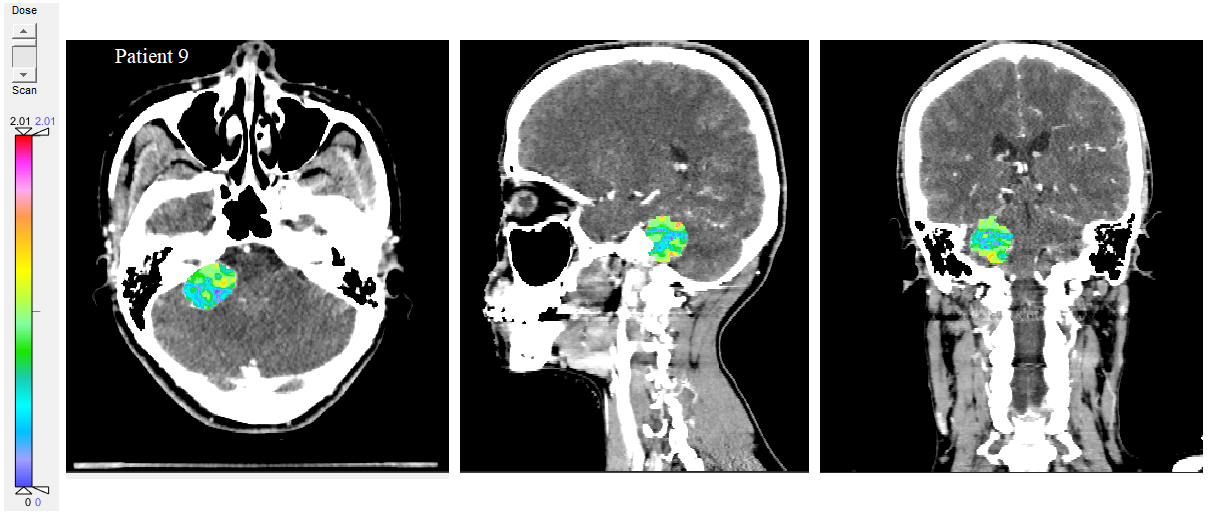

Supplement: Supplementary Appendix A — The target gamma difference for each patient. [file DataSheet_1.zip › Appendix A The target gamma difference for each patient/Patient 9-1.tif]
